# Pediatric HIV Infection and Decreased Prevalence of OPV Point Mutations Linked to Vaccine-associated Paralytic Poliomyelitis

**DOI:** 10.1093/cid/ciy635

**Published:** 2018-10-30

**Authors:** Meira S Halpern, Jonathan Altamirano, Yvonne Maldonado

**Affiliations:** Stanford School of Medicine, Stanford University, California

**Keywords:** OPV, HIV, VAPP, shedding, infants

## Abstract

**Background:**

Mutations associated with prolonged replication of the attenuated polioviruses found in oral poliovirus vaccine (OPV) can lead to vaccine-derived poliovirus (VDPV) and cause paralysis indistinguishable from that caused by wild poliovirus. In response, the World Health Organization has initiated the transition to exclusive use of inactivated poliovirus vaccine (IPV), with OPV administration in cases of outbreak. However, it is currently unclear how IPV-only vaccination, well known to provide humoral but not mucosal immunity, will impact the development of paralysis causing OPV variants. Children infected with human immunodeficiency virus (HIV) have been documented to show decreased mucosal immunity following OPV vaccination. Thus, HIV-infected children vaccinated with OPV may serve as proxy for children with IPV-only vaccination.

**Methods:**

We conducted a prospective study of Zimbabwean infants receiving OPV as part of their routine vaccination schedule. Stool samples collected from OPV-vaccinated children serially until age 24 months were tested for OPV serotypes using a real-time polymerase chain reaction protocol that quantifies the amount of mutant OPV variants found in each sample.

**Results:**

Out of 2130 stool samples collected from 402 infants 365 stool samples were OPV positive: 313 from 212 HIV-noninfected (HIV−) infants and 52 from 34 HIV-infected (HIV+) infants. HIV− infants showed significantly higher proportions of OPV mutants when compared to HIV+ infants.

**Conclusions:**

HIV infection is associated with a reduced proportion of OPV vaccine associated paralytic polio mutants. These results suggest that OPV administered to individuals previously vaccinated only with IPV will show decreased propensity for OPV mutations.

Since the Global Polio Eradication Initiative’s inception in 1988, paralysis due to wild poliovirus (WPV) has decreased by >99%, from an estimated 350000 cases in 1988 to 22 reported cases in 2017 [1, 2]. With eradication of WPV serotype 2 declared in 2015 and no WPV serotype 3 detected since November 2012, only WPV serotype 1 is believed to still be in circulation, with cases identified during 2017 only in Pakistan and Afghanistan [1, 3]. Thus, the goal of global polio eradication appears to be close.

The success of polio eradication efforts is largely due to the widespread use of Sabin oral poliovirus vaccine (OPV). OPV is particularly useful in low- and middle-income settings due to its ease of administration, low cost, and provision of community immunization coverage via fecal–oral transmission to unvaccinated household and community contacts [4]. However, continued use of OPV may now complicate eradication efforts because of the potential for continued circulation of OPV and development of OPV variants known to cause polio, including those related to vaccine-associated paralytic polio (VAPP) and vaccine-derived polioviruses (VDPV). Rapidly occurring canonical point mutations lead to VAPP, which is estimated to cause 2–4 cases/1000000 live births per year in countries using OPV [5]. In addition, long-term replication and mutation of OPV can lead to genetically divergent VDPVs, defined as 1% divergence from the parent strain for OPV serotypes 1 and 3 (OPV-1 and OPV-3) and 0.6% divergence from the parent strain for OPV serotype 2 (OPV-2) [6, 7]. Circulating VDPVs (cVDPVs), those with evidence of community transmission, are phenotypically indistinguishable from WPV, causing paralysis in children [6]. In 2017, 91 cases of cVDPVs were identified, >4 times more than the number of WPV cases [2].

The factors involved in the evolution of OPV into VAPP or VDPVs are not well characterized. Most data regarding VDPVs are derived from OPV isolates obtained from stool samples collected during investigations triggered by acute flaccid paralysis cases or detection of poliovirus in environmental samples after mutations have already accrued [8–13]. However, VAPP mutations have been well documented. These rapidly occurring point mutations occur in the internal ribosomal entry site located in the 5’ untranslated region of the poliovirus genome and are canonical point mutations specific to each OPV serotype (OPV-1, 480 G to A; OPV-2, 481 A to G; OPV-3, 472 U to C) [14–17]. The relationship, if any, of VAPP and VDPVs is unclear, but it is possible that both OPV variants are impacted by factors that affect intestinal replication of polioviruses, including intestinal immunity.

One consideration in understanding the evolution of OPV variants such as VAPP and VDPVs is the impact that pediatric infection with the human immunodeficiency virus (HIV) might have in the development of these OPV variants. In contrast to adults, children with HIV infection may manifest acquired humoral immunodeficiency and, as a result, decreased antibody responses to infections as well as to vaccines, including OPV [18, 19]. We and others have identified decreased seroconversion to OPV among children with HIV infection, as well as OPV shedding independent of the number of administered doses of the vaccine. However, we have not identified any significant impact of HIV infection on prolonged shedding or household transmission of OPV. The impact of HIV infection on development of OPV variants is important since there are more than 3 million children living with HIV, most in sub-Saharan Africa, where OPV remains the predominantly used polio vaccine [20–22].

In order to understand the association between pediatric HIV infection and development of OPV variants, we analyzed stool samples for the presence of OPV VAPP in a prospective study of HIV-infected (HIV+) and -uninfected (HIV–) Zimbabwean infants who received OPV as part of their routine childhood immunization. We chose to study development of VAPP mutations because they are rapidly occurring and therefore can be observed by following samples collected over a relatively short period of time.

## METHODS

### Study Design

In a previously published prospective cohort study conducted during 2008–2011, we recruited families with infants receiving their primary OPV vaccination series in Chitungwiza and Harare, Zimbabwe. Zimbabwean immunization guidelines during the study period included trivalent OPV administration to all infants/children at ages 3, 4, 5, and 18 months. OPV was also administered during supplementary immunization campaigns on 6–18 June 2009, 30 November–12 December 12 2009, and 24 May–1 June 2010. Infants were initially enrolled around age 3 months; subsequently, additional infants and young children were enrolled up to age 18 months to increase the study sample size. Stool samples were collected from infants and family members at planned visits when the enrolled infant was aged 3, 4, 5, 6, 9, 12, 18, 19, and 24 months. Blood samples were collected from infants at 5 study visits for testing (at age 3, 5, 9, 18, and 24 months), including HIV polymerase chain reaction (PCR) at each time point and enzyme-linked immunosorbent assay at age 18 months in order to confirm each infant’s HIV status. At each visit, the caregiver completed a questionnaire detailing the infant’s health and vaccination history. Stool samples were then processed and stored in our laboratory in Harare prior to shipment to our laboratory at Stanford University (California), where real-time (RT)-PCR was used to detect the presence of OPV serotypes using previously described methods [20]. For this study, we extracted information from the OPV isolates regarding the presence of OPV VAPP serotypes and determined the revertant proportion of OPV-1, -2, and -3 in each isolate as described in the Statistical Analyses section.

The analyses described here focus on comparing the HIV+ and HIV– infants with respect to the proportion of VAPP in their OPV-positive samples. All the stool samples from HIV+ infants and randomly age-matched samples from HIV– infants were tested for the presence of OPV. All the OPV-positive samples from infants with complete vaccination records and confirmed HIV status, collected at least 1 day after the first OPV dose, are included in the current study.

The Medical Research Council of Zimbabwe, the Research Council of Zimbabwe, and the Stanford University School of Medicine Institutional Review Board approved this study. All caretakers of the infants provided informed consent.

### Stool Assays

After collection, stool samples were stored at −80°C in our laboratory in Harare and then batched and shipped to our laboratory at Stanford University where they were stored at −80°C. The samples underwent RNA extraction, reverse transcription, and RT-PCR with assays designed to detect serotype-specific point mutations associated with VAPP serotypes. Proportions of revertant and nonrevertant OPV-1, -2, and -3 per isolate were calculated, as previously published and briefly described in the Statistical Analyses section [23]. We used a Biorad CFX384 Real-Time System and a 400 Reactive Florescence Units (RFU) threshold for detecting fluorescence.

### Statistical Analyses

**Figure 1.**
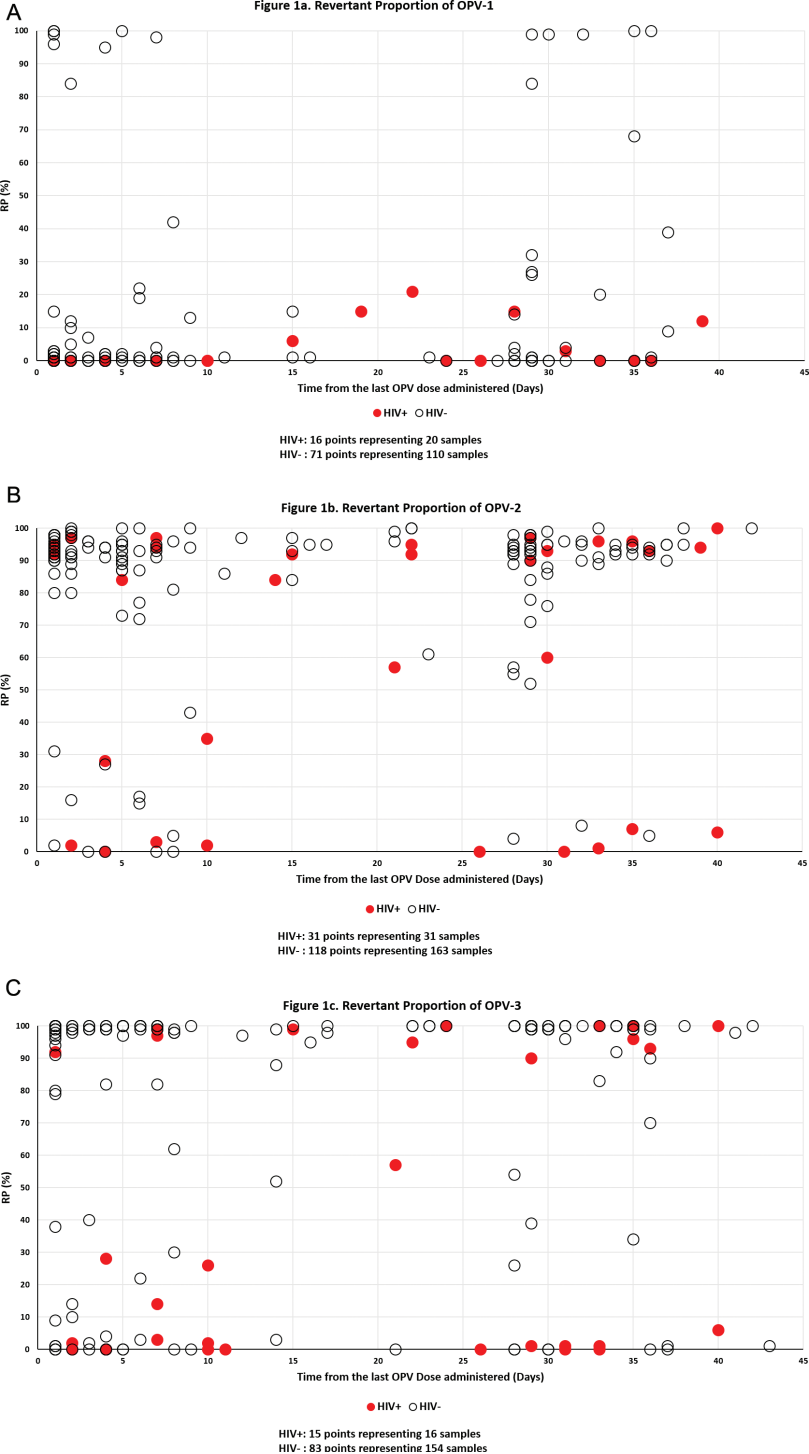
Revertant proportion (RP) of isolates in stool collected 1 to 42 days post oral poliovirus vaccination (OPV) from Zimbabwean vaccinated infants for OPV-1, -2, and -3. . Multiple isolates may have the same RP at the same time point post-vaccination in Figures 1*A*–1*C*, and the corresponding points in the graph cover each other. This is particularly true for isolates from human immunodeficiency virus uninfected (HIV–) infants. Major overlapping points for HIV– children: OPV-1: 25 nonrevertant isolates ≤14 days from vaccination; 9 nonrevertant isolated >21 days from vaccination. OPV-2: 33 revertant isolates ≤14 days from vaccination; 12 revertant isolated >21 days from vaccination. OPV-2: 41 revertant isolates ≤14 days from vaccination; 25 revertant isolated >21 days from vaccination. For more detail regarding all overlapping points, please see Supplementary Table S1.

The proportion of revertant OPV in each isolate, or revertant proportion (RP, from 0%–100%), was estimated using the following calculation: 2^−rev*CT*^/(2^−rev*CT*^ + 2^−nonrev*CT*^), where rev*CT* and nonrev*CT* are revertant and nonrevertant thresholds-crossing cycle numbers, respectively. As the RP distribution was heavily weighted to be either close to 100% or to 0% (Figure 1), we defined the samples as revertant if the RP of the isolate was >50%, and nonrevertant if it was ≤50%. Time from the last OPV dose was divided into 4 groups: 1–3, 4–21, 22–42, and >42 days. Because of the small sample size, the number of OPV doses prior to stool sample collection was combined as follows: first dose (OPV naive) and ≥2 doses (OPV exposed). The results describe all the samples included in the study.

Statistical analysis was conducted using SAS 9.3. Categorical data were compared using 2-tailed Fisher exact test. To avoid bias resulting from multiple samples from 1 infant, 1 sample per person was chosen for statistical testing. This was done in 2 ways. In the first process, one sample per person was chosen, either from the samples collected after 3 or more OPV doses or, if there were none, the samples collected after the largest number of OPV doses available. The earliest of multiple samples picked by these criteria was used. In the second process, 1 sample per infant was randomly picked. The process was repeated 500 times, and the means of the results of the statistical tests were calculated. A *P* value of ≤.05 was considered statistically significant.

## RESULTS

A total of 421 infants were enrolled in the study, 19 of whom were excluded because of unclear HIV status (11), missing OPV information (5), or failure to submit stool samples (3). Of the 402 included infants, 92 were HIV+ and 310 were HIV–. A total of 2130 useable stool sample were collected post OPV vaccination from the included infants, 80% of which (77% and 100% from HIV– and HIV+ infants, respectively) were tested for poliovirus shedding, which was found in 365 of the samples. These 365 stool samples, from 246 infants, are included in the presented analysis: 313 samples from 212 HIV– infants and 52 samples from 34 HIV+ infants. A total of 61% of the infants in the study had only 1 positive sample; 64% of the samples were positive for 1 Sabin type only.

The overall proportion of revertant samples, per their definition in the Methods section, is associated with the OPV serotype: 17% of 139 OPV-1–positive samples, 87% of 215 OPV-2–positive samples, and 74% of 175 OPV-3–positive samples. Of the 32 positive samples of all Sabin types collected >42 days from vaccination, 88% were revertant (15 and 13 samples from HIV+/− infants, respectively; 27 samples with RP ≥95% and 1 sample RP = 84%). One sample, collected 43 days from the second OPV dose from an HIV– infant, was revertant to OPV-2 and nonrevertant to OPV-3. Only 3 OPV-positive samples collected >43 days from vaccination were nonrevertant; 2 were collected from HIV+ infants 70 and 115 days from the first and third OPV doses and contained only nonrevertant OPV-2; 1 sample collected during a supplementary immunization campaign from an HIV– infant 89 days after his third OPV dose had RP = 7% for OPV-1. RP values as a function of time from vaccination for all 3 OPV serotypes in samples collected from HIV+/− infants on days 1–42 from vaccination are presented in Figure 1A–1C. The figure demonstrates that the RP of OPV isolates is lower for HIV+ infants than for HIV– infants. The mean RP for all OPV isolates collected within 42 days from vaccination by OPV type and for HIV– vs HIV+ infants were OPV-1–16% vs 4%, OPV-2–84% vs 61%, and OPV-3–77% vs 34%. The overall proportions of samples with majority revertant mutations collected within 42 days from vaccination by OPV type were 14%, 91%, and 78% for HIV– infants and 0%, 65%, and 31% for HIV+ infants (Table 1).

**Table 1.** Proportion of Revertant Samples Among All Positive Stool Samples Collected in 42 Days From Vaccination, by Polio Serotype, Time from the Last Prestool Oral Poliovirus Vaccine Dose, and Human Immunodeficiency Virus Status A. OPV-1

		All Positive Samples by Time from OPV						All Positive Samples Collected in 42 Days from OPV				
		≤3 Days from Vaccination		4–21 Days from Vaccination		22–42 Days from Vaccination		All Records		One Record per Infant		
		N (%)^a^	% Revertant	N (%)^a^	% Revertant	N (%)^a^	% Revertant	N	% Revertant	N^	% Revertant	*P* Value
OPV naive (1 dose)	HIV–	5 (17)	**0**	3 (10)	**0**	22 (73)	**9**	30	**7**	30	**7**	1
	HIV+	0 (0)	**…**	4 (57)	**0**	3 (43)	**0**	7	**0**	7	**0**	
OPV exposed (≥2 doses)	HIV–	32 (40)	**16**	32 (40)	**9**	16 (20)	**31**	80	**16**	73	**18**	.2
	HIV+	3 (23)	**0**	3 (23)	**0**	7 (54)	**0**	13	**0**	11	**0**	
All	HIV–	37 (34)	**14**	35 (32)	**9**	38 (35)	**18**	110	**14**	101	**14**	.2
	HIV+	3 (15)	**0**	7 (35)	**0**	10 (50)	**0**	20	**0**	17	**0**	

^Multiple samples from the same infant: HIV–: 7 with 2 samples, 1 with 3 samples; HIV+: 3 with 2 samples.

^a^ Percent of all samples collected in 42 days from vaccination.

**Table:** B. OPV-2

		All Positive Samples by Time from OPV						All Positive Samples Collected in 42 days from OPV				
		≤3 Days from Vaccination		4–21 Days from Vaccination		22–42 Days from Vaccination		All Records		One Record per Infant		
		N (%)^a^	% Revertant	N (%)^a^	% Revertant	N (%)^a^	% Revertant	N	% Revertant	N^	% Revertant	*P* Value
OPV-naive (1 dose)	HIV–	8 (15)	**100**	6 (11)	**83**	40 (74)	**100**	54	**98**	54	**98**	.001
	HIV+	0 (0)	...	3 (33)	**33**	6 (67)	**67**	9	**56**	9	**56**	
OPV exposed (≥2 doses)	HIV–	46 (42)	**91**	41 (38)	**86**	22 (20)	**83**	109	**87**	95	**87**	.01
	HIV+	4 (18)	**75**	8 (36)	**63**	10 (45)	**70**	22	**68**	18	**61**	
All^a^	HIV–	54 (33)	**93**	47 (29)	**83**	62 (38)	**95**	163	**91**	142	**92**	<.0001
	HIV+	4 (13)	**75**	11 (35)	**55**	16 (52)	**68**	31	**65**	25	**56**	

^Multiple samples from the same infant: HIV–: 14 with 2 samples, 2 with 3 samples, 1 with 4 samples; HIV+: 4 with 2 samples, 1 with 3 samples.

^a^ Percent of all samples collected in 42 days from vaccination.

**Table:** C. OPV-3

		All Positive Samples by Time from OPV						All Positive Samples Collected in 42 days from OPV				
		≤3 Days from Vaccination		4–21 Days from Vaccination		22–42 Days from vaccination		All Records		One Record per Infant		
		N (%)^a^	% Revertant	N (%)^a^	% Revertant	N (%)^a^	% Revertant	N	% Revertant	N^	% Revertant	*P* Value
OPV naive (1 dose)	HIV–	2 (8)	**100**	1 (4)	**0**	22 (88)	**77**	25	**76**	25	**76**	.07
	HIV+	0 (0)	...	4 (67)	**25**	2 (33)	**50**	6	**33**	6	**33**	
OPV exposed (≥2 doses)	HIV–	51 (40)	**76**	44 (34)	**75**	34 (26)	**85**	129	**78**	111	**79**	.001
	HIV+	1 (10)	**0**	4 (40)	**25**	5 (50)	**40**	10	**30**	9	**22**	
All^a^	HIV–	53 (34)	**77**	45 (29)	**73**	56 (36)	**82**	154	**78**	132	**80**	.0001
	HIV+	1 (6)	**0**	8 (50)	**25**	7 (44)	**43**	16	**31**	15	**27**	

^Multiple samples from the same infant: HIV–: 20 with 2 samples, 1 with 3 samples; HIV+: 1 with 2 samples.

^a^ Percent of all samples collected in 42 days from vaccination.

Notes for Tables 1A–1C: The presented *P* value was estimated based on a selection of 1 sample per infant in a category according to the following rule: (i) pick the sample collected after the third OPV dose; (ii) if none in (i), pick the sample with the largest number of prestool OPV doses; (iii) if more than 1 sample satisfies (i) or (ii), pick the earliest among them. Randomly choosing 1 sample per infant and repeating the process 500 times yields mean *P* values very close to the ones presented. The quantities that stand in comparison are in bold.

In the column of 1 record per person, the sum of N in the rows for 1 OPV dose and ≥2 OPV doses do not sum up to N in the All row because some infants had 2 samples, 1 after dose 1 and 1 after a later dose. Both are used in the first and second rows, but only 1 of them is picked in row 3.

Abbreviations: HIV+, human immunodeficiency virus infected; HIV−, human immunodeficiency virus uninfected; OPV, oral poliovirus vaccine.

Table 1 summarizes the proportion of revertants among all positive samples collected within 42 days after vaccination. For all OPV serotypes, the proportion of revertant samples is higher in HIV– infants than in HIV+ infants. This is apparent for all serotypes and in most of the presented time intervals, irrespective of the number of prestool OPV doses. The only exception is OPV-3 in 4–22 days from the first OPV dose, where there was only 1 positive sample from HIV– infants. The differences in the proportion of revertant samples between HIV– and HIV+ are large and highly statistically significant for both OPV-2 and OPV-3, both having high proportions of revertant samples. The difference between HIV+ and HIV– infants in the proportion of OPV-1 revertant samples is not statistically significant.

## DISCUSSION

Our main finding in this study is the clear association between reduced OPV VAPP reversion and pediatric HIV infection based on OPV isolates collected after the administration of routine OPV vaccination. To study that association, we estimated the revertant proportion in 331 stool samples from 212 HIV– infants and 52 samples from 34 HIV+ infants, all with evidence of OPV shedding. We found that overall shedding >2 months from vaccination (31 isolates) was predominantly VAPP. However, 2 of the samples collected from HIV+ infants were still predominantly nonrevertant OPV-2 >2 months post-vaccination, which may also represent community acquisition and shedding of OPV. Previously, we demonstrated [24, 25] the rapid reversion of OPV-3 in HIV– infants. In this study we show that OPV-2 reverts even more rapidly than OPV-3, while OPV-1 reverts much more slowly. However, for all OPV serotypes, reversion among HIV+ infants lags considerably behind reversion among HIV– infants. For OPV-2 and OPV-3, the gap is highly statistically significant. However, establishing statistical significance for the 0% vs 14% OPV-1 reversion proportion of HIV+ and HIV– infants requires a larger sample size than that available in the current study.

Our previously published study with this cohort [20] showed a decrease in OPV shedding after ≥3 OPV doses in HIV– infants, but not in HIV+ infants, as well as that HIV+ infants were also less likely to seroconvert after a comparable number of OPV doses as HIV– infants and produce significantly lower polio geometric mean titers. This suggests that HIV+ infants, shown in that study to produce poor humoral polio immunity, may also produce suboptimal mucosal immunity to OPV. The reduced OPV reversion in HIV+ children may be the result of this deficit in mucosal immunity. Mucosal immunity resulting from repeated OPV exposure and development of humoral and mucosal immunity could provide selective pressure in the gut for OPV to mutate.

Our study is unique in its use of a sensitive method to distinguish revertant and nonrevertant OPV-1, -2, and -3 [23]. Using our protocol, we were able to analyze multiple samples from vaccinated children to assess the difference in the revertant proportion between HIV+ and HIV– children. With more than 3 million children living with HIV globally, understanding how OPV impacts this population is vital. Further, as HIV+ children fail to develop mucosal immunity to OPV, this data might serve as a proxy for other populations without mucosal immunity, including individuals with other immunodeficiency disorders or OPV-naive children.

The current eradication policy calls for OPV vaccination campaigns in the event of polio outbreaks [26, 27]. Studies have demonstrated that IPV alone produces limited mucosal immunity to poliovirus [28, 29], which is also the case for HIV+ infants [20] whose OPV shedding is independent of the number of prior OPV doses administered. Our current study demonstrates reduced OPV mutation probability in HIV+ infants. These results suggest the likelihood that increased shedding of OPV in the presence of decreased mucosal immunity, such as that found in HIV–infected infants and after IPV-only vaccination, may result in decreased OPV mutations, at least for canonical VAPP mutation. Whether or not these results can be extended to healthy children with IPV-induced immunity to polioviruses or to decreased VDPV mutations is not known.

A limitation of our study is the relatively small number of samples from HIV+ infants. Under the original study design, HIV+ and HIV– pregnant women were recruited with the aim that the resulting ratio of HIV+ to HIV– infants would be 1:2. With mother-to-child HIV transmission much reduced by successful HIV prevention programs and increased uptake of antiretroviral therapy in Zimbabwe during the study period, few recruited babies were diagnosed as HIV+. The number of samples collected from HIV+ infants was further reduced by the increased mortality among HIV+ children when compared to HIV– children; 14% vs 2% in this study.

Another limitation of our study is our inability to distinguish between primary OPV shedding after an OPV dose and secondary shedding due to previous OPV doses or acquisition of OPV from community transmission. However, because of the relatively large number of samples obtained early after each vaccination and the large number of patients enrolled, it is likely that the majority of OPV-positive isolates were related to primary vaccine shedding rather than community acquisition. Moreover, there is no evidence to suggest association between the probability of secondary vaccine shedding and HIV infection.

In this study we have shown that the revertant proportion of OPV shed in stool following OPV vaccination is lower in HIV+ infants, whose mucosal immunity is poor, than in HIV– infants. The similarities in mucosal immunity in HIV+ and OPV-naive infants suggest that the decreased selective pressure on OPV to mutate may also exist in OPV-naive infants. As a result, OPV-naive children may also demonstrate decreased reversion of OPV following OPV challenge. Additional studies of OPV mutations among IPV-vaccinated infants and children could further elucidate the impact of IPV in reversion of OPV and development of OPV variants.

## Supplementary Data

Supplementary materials are available at *Clinical Infectious Diseases* online. Consisting of data provided by the authors to benefit the reader, the posted materials are not copyedited and are the sole responsibility of the authors, so questions or comments should be addressed to the corresponding author.

## Supplementary Material

Supplemary_Table_S1

## Notes

***Acknowledgments*.** The authors thank the study nurses, study counselors, Zimbabwe AIDS Prevention Project (ZAPP) data manager (Lovemore Chinyere), Drs Stephanie Troy Nivedita Srinivas and Marisa Holubar, as well as ChunHong (Stacy) Huang for obtaining and analyzing the samples; Dr LauraLe Dyner, Dr Micah Simoyi, and the Chitungwiza Health Department; Professors Godfrey Woelk and Simbarashe Rusakaniko along with the ZAPP-University of Zimbabwe (UZ) support staff; the UZ College of Health Sciences; the Zimbabwe National Poliomyelitis Reference Laboratory; and especially all the mothers and infants who participated in the study.

The authors acknowledge the late Professor Isidore-Evans Pazvakavambwa, who helped design the study, and the late Professor Michael Chirara, who advised on the laboratory assays.

***Financial support*.** This study was funded by the National Institutes of Health (R01 AI068577 to Y. A. M.).

***Supplement sponsorship.***  This article appears as part of the supplement “Polio endgame and beyond: Vaccine choices, transmission dynamics, and surveillance implications,” sponsored by The Bill & Melinda Gates Foundation.

***Potential conflicts of interest.*** All authors: No reported conflicts of interest. All authors have submitted the ICMJE Form for Disclosure of Potential Conflicts of Interest. Conflicts that the editors consider relevant to the content of the manuscript have been disclosed.
