# Supplementary material for: Pediatric HIV Infection and Decreased Prevalence of OPV Point Mutations Linked to Vaccine-associated Paralytic Poliomyelitis
Source: Clin Infect Dis. 2018 Oct 30;67(Suppl 1):S78–84. doi: 10.1093/cid/ciy635 (PMC6206102; doi:10.1093/cid/ciy635)
Supplement: Supplemary_Table_S1 [file ciy635_suppl_supplemary_table_s1.docx]

**Table S1. Number of overlapping points for HIV- and HIV+ infants**

|  | | Overlapping Points for HIV- Infants | | Overlapping Points for HIV+ Infants | |
| --- | --- | --- | --- | --- | --- |
|  |  | ≤14 d. from vaccination | >21 d. from vaccination | ≤14 d. from vaccination | >21 d. from vaccination |
| OPV-1 | Nonrevertant (N) | 25 | 9 | 2 | 2 |
|  | Revertant (N) | 1 | 0 | 0 | 0 |
| OPV-2 | Nonrevertant (N) | 0 | 0 | 0 | 0 |
|  | Revertant (N) | 33 | 12 | 0 | 0 |
| OPV-3 | Nonrevertant (N) | 5 | 2 | 1 | 0 |
|  | Revertant (N) | 41 | 25 | 0 | 0 |

Table S1 Note. No overlapping points between days 15 – 21. As a result, this range is excluded from the table.
